# Supplementary material for: Highly Efficient Blood Protein Analysis Using Membrane Purification Technique and Super-Hydrophobic SERS Platform for Precise Screening and Staging of Nasopharyngeal Carcinoma
Source: Nanomaterials (Basel). 2022 Aug 8;12(15):2724. doi: 10.3390/nano12152724 (PMC9370769; doi:10.3390/nano12152724)
Supplement: Supplementary file 1 [file nanomaterials-12-02724-s001.zip › nanomaterials-1793384-supplementary.pdf]

supplementary materials

Highly Efficient Blood Protein Analysis Using Membrane Purification Technique and Super-Hydrophobic SERS Platform for Precise Screening and Staging of Nasopharyngeal Carcinoma

Jinyong Lin [1,2,†], Youliang Weng [2,†], Xueliang Lin [3,†], Sufang Qiu [2], Zufang Huang [1], Changbin Pan [1], Ying Li [2], Kien Voon Kong [4], Xianzeng Zhang [1,*] and Shangyuan Feng [1,*]

1 Key Laboratory of OptoElectronic Science and Technology for Medicine, Ministry of Education, Fujian Provincial Key Laboratory for Photonics Technology, Fujian Normal University, Fuzhou 350007, China
2 Clinical Oncology School of Fujian Medical University, Fujian Cancer Hospital, Fuzhou 350014, China
3 Fujian Provincial Key Laboratory for Advanced Micro-Nano Photonics Technology and Devices, Research Center for Photonics Technology, Quanzhou Normal University, Quanzhou 362046, China
4 Department of Chemistry, National Taiwan University, Taipei 10617, Taiwan
* Correspondence: xzzhang@fjnu.edu.cn (X.Z.); syfeng@fjnu.edu.cn (S.F.)
† These authors contributed equally to this work.

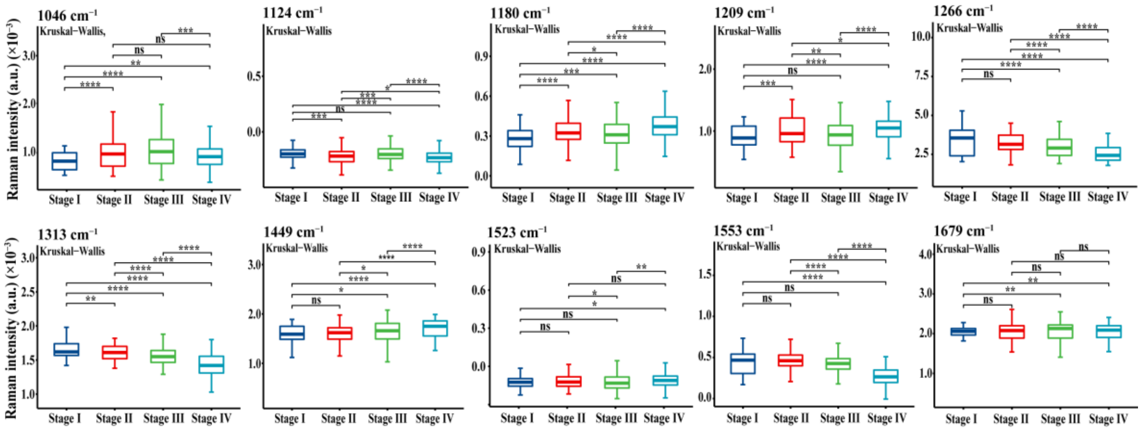

Figure S1 Box plots of others of the protein SERS peak intensities for NPC subjects at each of the four stages. Abbreviations: ns indicates no significance; *$p < 0.05$; **$p < 0.01$; ***$p < 0.001$; ****$p < 0.0001$ (Kruskal–Wallis test).
